# Genome–Transcriptome Transition Approaches to Characterize Anthocyanin Biosynthesis Pathway Genes in Blue, Black and Purple Wheat

**DOI:** 10.3390/genes14040809

**Published:** 2023-03-27

**Authors:** Payal Kapoor, Saloni Sharma, Apoorv Tiwari, Satveer Kaur, Anita Kumari, Humira Sonah, Ajay Goyal, Meena Krishania, Monika Garg

**Affiliations:** 1 National Agri-Food Biotechnology Institute, Mohali 140308, India; 2 Chitkara University, Himachal Prades, Kalujhinda, Baddi 174103, India; 3 Center of Innovative and Applied Bioprocessing, Mohali 140308, India

**Keywords:** Anthocyanins, genes, color wheat, Anthocyanin biosynthesis, phenylpropanoid, black wheat

## Abstract

Colored wheat has gained enormous attention from the scientific community, but the information available on the anthocyanin biosynthetic genes is very minimal. The study involved their genome-wide identification, in silico characterization and differential expression analysis among purple, blue, black and white wheat lines. The recently released wheat genome mining putatively identified eight structural genes in the anthocyanin biosynthesis pathway with a total of 1194 isoforms. Genes showed distinct exon architecture, domain profile, regulatory elements, chromosome emplacement, tissue localization, phylogeny and synteny, indicative of their unique function. RNA sequencing of developing seeds from colored (black, blue and purple) and white wheats identified differential expressions in 97 isoforms. The *F3H* on group two chromosomes and *F3′5′H* on 1D chromosomes could be significant influencers in purple and blue color development, respectively. Apart from a role in anthocyanin biosynthesis, these putative structural genes also played an important role in light, drought, low temperature and other defense responses. The information can assist in targeted anthocyanin production in the wheat seed endosperm.

## 1. Introduction

Anthocyanins are water-soluble pigments of the phenylpropanoid class that give red to purple hues to flowers, seeds, fruits, leaves and stems, make the appearance attractive and assist in roles such as pollination and seed dispersal. They also play an important role in abiotic and biotic stressors. Anthocyanins are also advantageous to human health due to their antioxidant potential that may protect against heart disease, cancer and other chronic diseases [1]. In order to address the growing demand for health-promoting components in our daily diet, a thorough understanding of anthocyanin biosynthesis is required. Researchers have studied the anthocyanin biosynthetic pathway well and characterized the corresponding genes in various plant species. *Arabidopsis thaliana* has served as a model plant for the last two decades to study anthocyanins’ biosynthesis, regulation and transport. Most structural genes have been identified and functionally characterized in it. These studies have contributed to the comprehensive understanding of anthocyanin biosynthesis and revealed the accumulation and metabolic profiles of anthocyanins in *A. thaliana* [2]. Anthocyanins are synthesized from three molecules of malonyl CoA derived from fatty acid metabolism and one of p-coumaroyl CoA synthesized from phenylalanine via the general phenylpropanoid pathway. The enzymes evolved in anthocyanin biosynthesis are as follows: phenylalanine ammonia-lyase (PAL), cinnamate 4-hydroxylase (C4H), 4-coumarateCoA ligase (4CL), chalcone synthase (CHS), chalcone isomerase (CHI), flavanone 3-hydroxylase (F3H), flavonoid 3′-hydroxylase (F3′H), flavonoid 3′5′-hydroxylase (F3′5′H), dihydroflavonol 4-reductase (DFR), anthocyanidin synthase (ANS)/leucoanthocyanidin dioxygenase (LDOX) and uridine phosphate-glucose: flavonoid-O-glycosyltransferase (UFGT). 

The general phenylpropanoid pathway initiates with *PAL*, *C4H* and *4CL*, giving rise to cinnamate, p-coumarate and p-coumaroyl coA, respectively, that serve as the foundation for all subsequent branches and metabolites [3]. The anthocyanin biosynthesis genes are categorized into structural and regulatory genes. The structural genes encode the enzymes associated with the formation of colored anthocyanin compounds [4]. The second set of genes encodes for regulatory components that regulate structural gene expression, which is primarily aided and abetted by complexes formed by myeloblastosis (MYB) and basic helix-loop-helix (bHLH) transcription factors that include WDR (WD40 repeats) proteins [5]. The anthocyanin biosynthesis is initiated with CHS catalyzing the stepwise condensation of three molecules of malonyl-CoA with one molecule of 4-coumaroyl-CoA to form the basic structure of flavonoids (tetra hydroxychalcone and naringenin chalcone), which is rapidly isomerized to the colorless naringenin by CHI. Naringenin is then converted to dihydroflovnol by F3H, F3′H and F3′5′H. In the first step of dihydroflovnol synthesis, F3H converts naringenin to dihydrokaempferol, which can be hydroxylated on the 3′ or 5′ positions of the B-ring by F3′H to produce dihydroquercetin or by F3′5′H to form dihydromyricetin. DFR is a specific enzyme for anthocyanin synthesis. It catalyzes the leucoanthocyanidin production from dihydroflovnols. Subsequently, LDOX/ANS converts leucoanthocyanidins into anthocyanidins. Anthocyanins are further glycosylated by UFGT [6]. Biochemical approaches have demonstrated that all anthocyanin pigments are derived from one of three aglycones: pelargonidin, cyanidin and delphinidin. The main determinants of the apparent color of these pigments are the hydroxylation and methylation patterns and the number and type of sugars on the β ring of the flavonoid molecule [7].

The anthocyanin biosynthesis pathway is still obscure in wheat due to the complex nature of the genome, and this might be because color wheat has been poorly researched until recently [8,9]. To date, very few researchers have tried to understand anthocyanin biosynthetic genes in wheat. In wheat, *CHS* has been isolated and characterized with a molecular weight and isoelectric point of approximately 41 kDa and 5, respectively, by Wu et al. [10]. Six copies of CHS genes have been mapped on chromosomes 1A, 1B, 1D, 2A, 2B and 2D in wheat [11]. The CHS enzyme is a monomer and composed of ~220 amino acid residues. Only three differentially expressed F3H genes were identified and assigned to the long arm of chromosome 2A, 2B and 2D in red wheat [12] and one *F3H* was recognized at the 2A chromosome of purple wheat [13]. Three homologs of *DFR* have been characterized, i.e., TaDFR-A, TaDFR-B and TaDFR-D, and assigned to chromosome 3A, 3B and 3D, respectively, in both red [14] and purple wheat [15]. Until now, five copies of *ANS* have been identified, out of which two copies each were assigned to chromosomes 6A and 6B, respectively, and one copy was assigned to chromosome 6D in wheat [16]. One F3′5′H gene was identified in the 2AL chromosome of purple wheat [13], and one in the 4D chromosome of blue wheat [17]. 

Previously, the anthocyanin biosynthetic genes in *A. thaliana* and *Oryza sativa* were identified and analyzed by comparative genomic analysis, but this was not done in wheat. Understanding the genetic differences between anthocyanin rich wheat and white wheat (amber colored wheat) is important as it may reveal the differences in the genes encoding enzymes for the anthocyanin biosynthesis pathway. Thus, to understand the correlation between gene expression and anthocyanin accumulation, especially in colored wheat, we systematically identified and analyzed vital structural genes of anthocyanin biosynthesis in wheat. This study employed a bioinformatics approach to identify conserved domains, annotations and phylogeny of the anthocyanin biosynthesis genes in wheat. We also employed the comparative transcriptomics approach to study the expression patterns of the key structural genes of anthocyanin biosynthesis in colored wheat (black, blue and purple) and white wheat. The results of our studies will advance the understanding of anthocyanin biosynthesis at the gene expression level and provide a foundation for further wheat cultivar improvement and the breeding of novel cultivars of enhanced nutritional value. 

## 2. Results

### 2.1. In Silico Identification of Anthocyanin Biosynthesis Pathway Genes in Wheat

The anthocyanin biosynthesis pathway is part of the phenylpropanoid pathway and involves a series of enzymatic reactions catalyzed by a set of structural genes belonging to various enzyme superfamilies. After conducting the BlastP search of the eight structural genes having 37.2–92.6% identity with Arabidopsis and rice, 95 *CHS*, 7 *CHI*, 101 *F3H*, 491 *F3′H*, 51 *F3′5′H*, 133 *DFR*, 73 *ANS* and 242 *UFGT* were identified. The gene name, the number identified and the respective gene families have been given in Table 1.

**Figure 1:**
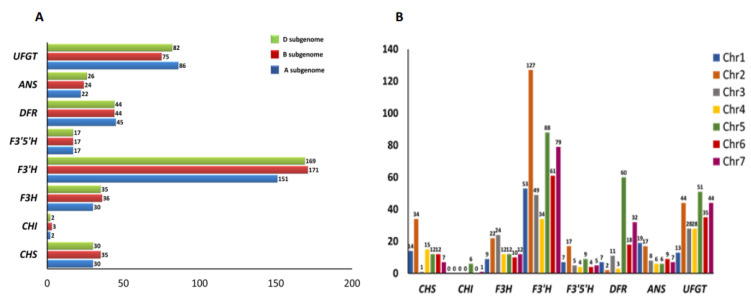
(**A**) Genomic and (**B**) chromosome distribution of genes of anthocyanin biosynthesis pathway in wheat.

The distribution of the key structural genes of the anthocyanin biosynthesis pathway in the wheat genome has been given in Figure 1A. It was observed that subgenomes B and D carried the maximum number of structural genes of the anthocyanin biosynthesis pathway, with 405 genes in each subgenome. Subgenome A had the least number, with 383 genes (Figure 1A). Among the seven chromosomes, chromosome two had the maximum number of structural genes of the anthocyanin biosynthesis pathway, and chromosome four had the least number.

Out of the 95 putative CHS genes, subgenomes A and D had an equal number of CHS genes, and chromosome two had the maximum number of CHS genes, while chromosome three had the least number (Figure 1B). The BlastP search identified seven CHI genes in the wheat genome, with subgenomes A and D having an equal number. CHI genes were mapped on chromosomes five and seven only, with chromosome five having the maximum number. Subgenome B had the maximum number of F3H genes, followed by subgenome D. Most of the F3H genes were mapped on chromosome three, followed by chromosome two, and chromosome one had the least number of F3H genes. The F3′H and F3′5′H genes were distributed mainly on chromosome two. F3′H genes were primarily distributed on subgenome B, followed by D and A, while the F3′5′H genes were distributed equally on subgenomes A, B and D. The DFR genes were almost equally distributed among the three subgenomes with the maximum number of genes on chromosome five and the least on chromosome two. The ANS gene had an almost equal distribution among the three subgenomes with the maximum number on chromosome 1. The UFGT genes were mainly present in subgenome A, followed by D and B, with the maximum number on chromosome five and the least on chromosome three.

### 2.2. Physiochemical Properties and Domain Analysis of Putative Anthocyanin Biosynthetic Genes in Wheat

The isoelectric point, molecular weight, subcellular localization and identified domains of the putatively identified key structural genes of the anthocyanin biosynthesis pathway in wheat have been tabulated in Table 2 below. The domains of these putative proteins were analyzed using the conserved domain database (CDD) tool hosted in NCBI and this revealed that CHS belonged to the chal_sti_synt domain, CHI belonged to the isomerase, and F3H and ANS belonged to the 2-oxoglutarate-dependent dioxygenase (2-ODD) superfamily. *F3′H* and *F3′5′H* belonged to the cytochrome p450 monooxygenase superfamily, and UFGT belonged to the Glycosyltransferase_GTB-type superfamily. *F3′H* and *F3′5′H* were the largest in size. *UFGT* had the highest isoelectric point compared to the other genes, and all were chloroplast-specific. Figure S1 shows the pictorial representation of the identified Pfam domains in key structural genes.

### 2.3. Comparative Transcriptomic Analysis of the Putative Anthocyanin Biosynthetic Pathway Genes in Colored Wheat

**Figure 2:**
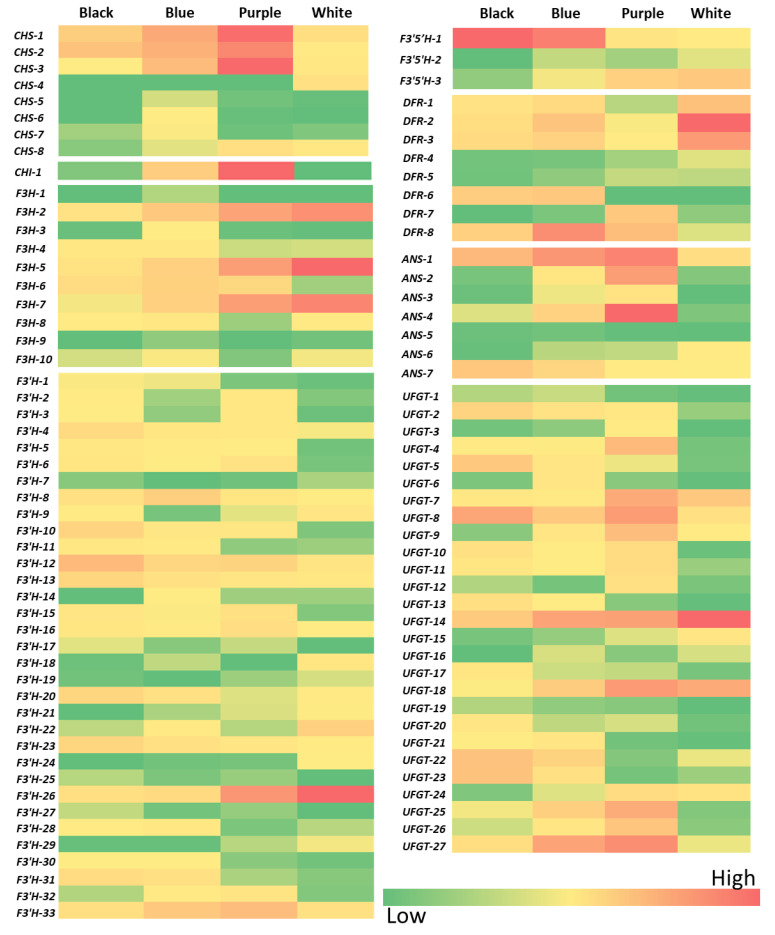
Heatmaps depicting the relative expression of studied genes in different colored wheat lines.

**Figure 3:**
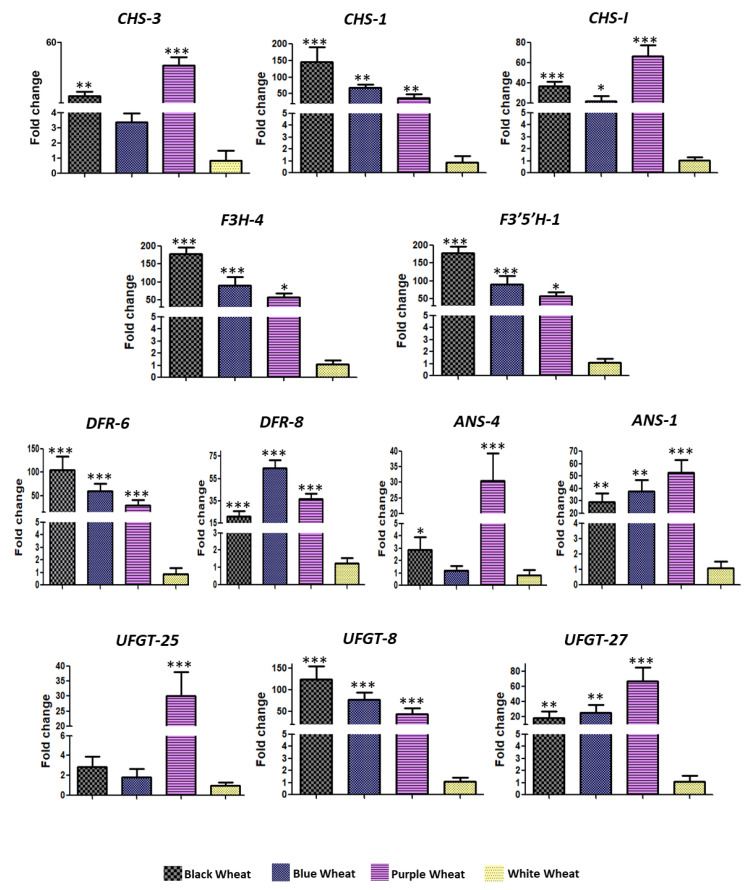
Quantitative real time PCR-based expression validation of selected genes. Significance level was represented by * depicting the *p*-value < 0.05, ** depicting the *p*-value < 0.001 and *** depicting the *p*-value < 0.0001 in comparison to white wheat.

The isoelectric point, molecular weight, subcellular localization and the expression values of the differentially expressed transcripts in the three different colors of wheat variety, i.e., purple, blue and black wheat vs. white wheat, were used in the search to deduce the expression of various target genes (CHS, CHI, F3H, F3′H, F3′5′H, DFR, ANS and UFGT). The expression pattern of the DEGs (with FDR < 0.05) pertaining to various target genes is depicted in the heatmaps in Figure 2 (Table S2). Out of the BlastP search, which identified 95 CHS genes, 8 genes were found to be differentially expressed among the colored wheat varieties. Subgenome A carried the maximum number of differentially expressed genes (DEGs) (five genes), and subgenomes B and D carried an equal number of differentially expressed genes, i.e., two. Out of the seven putative CHI genes, only one gene on chromosome five was expressed differently among the various wheat varieties. The number of differentially expressed F3H genes was 10 out of the 101 putative genes. Subgenome B carried the maximum number of F3H DEGs, followed by subgenomes A and D. Most of the DEGs were mapped onto chromosome two, followed by chromosomes seven and three. The number of DEGs in F3′H genes was 33 out of 491, mostly mapped on subgenome D and chromosome two. The number of DEGs in putatively identified F3′5′H genes was three, mapped on subgenome D, with one each on chromosomes one, two and five. The number of putatively identified DFR DEGs was eight, with the maximum and equal number found on subgenomes B and D and mostly mapped on chromosome three. Among putatively identified 74 ANS genes, the number of DEGs was seven, mostly mapped primarily on subgenome D and chromosome six. The number of DEGs in 243 putative UFGT genes was 27. Subgenomes A and B carried a maximum and an equal number of UFGT DEGs. The maximum number of UFGT DEGs was present on chromosome two and the minimum on chromosome three. Putative DEG isoforms also showed a variable expression pattern in all the four wheat lines. Surprisingly, some isoforms of all anthocyanin biosynthesis genes also showed expression in white wheat. Expression pattern of putative DEGs from each biosynthetic gene were validated through qRT -PCR (Figure 3). It has been observed that randomly selected isoforms of DEGS of respective biosynthetic genes showed similar patterns to transcriptome data. *TaCHS* and *TaCHI* were highly expressed in purple wheat, whereas *TaF3′H* and *TaF3′5′H* were highly expressed in blue and black wheat. In lateral biosynthetic genes, i.e., TaDFR, Ta*ANS* and Ta*UFGT*, different isoforms were observed to express in purple, blue and black wheat (Figure 2 and Figure 3). A higher expression of *CHS* isoforms, specifically the 2A and 2D isoforms, was observed in purple wheat compared to blue and black wheat. Furthermore, only one differentially expressed CHI gene was observed in colored wheat.

### 2.4. Genomic Distribution and Chromosomal Localization of the Putative Anthocyanin Biosynthesis Pathway Genes in Wheat

**Figure 4:**
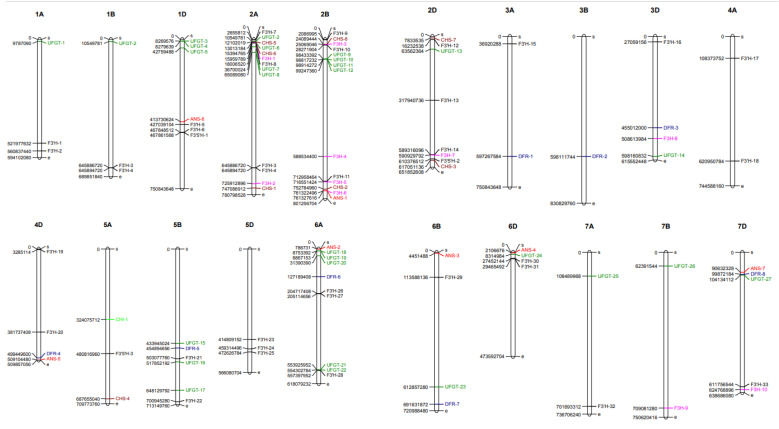
Chromosomal localization of anthocyanin biosynthetic DEG isoforms of the wheat genome.

The DEGs of key structural genes were mapped on the wheat genome (Figure 4). Of the seven CHS DEGs, chromosome 2A carried the majority, while chromosomes 2D and 5A carried the least and an equal number. The CHI DEG was present on chromosome 5A. Chromosome 2B harbored the highest number of F3H DEGs, while the chromosomes 2D, 3D, 7B and 7D harbored the least number. The 33 DEGs in F3′H genes were mainly mapped on chromosomes 2B, 2D, 5D and 6A, while the least and an equal number was mapped on chromosomes 3A, 3D, 6B, 7A and 7D. Three F3′5′H DEGs were equally distributed on chromosomes 1D, 2D and 5A of the wheat genome. Each DFR DEG was equally distributed on chromosomes 3A, 3B, 3D, 4D, 5B, 6A, 6B and 7D of the wheat genome. Each ANS DEG was mapped on chromosomes 1D, 2B, 4D, 6A, 6B, 6D and 7D of the wheat genome. Chromosome 6A harbored the highest number of UFGT DEGs, while the least number was found on chromosomes 1A, 1B, 2D, 3D, 6B, 6D, 7A, 7B and 7D (Figure 4).

### 2.5. Gene Structure and Motif Analysis of Differentially Expressed Putative Anthocyanin Biosynthesis Pathway Genes

**Figure 5:**
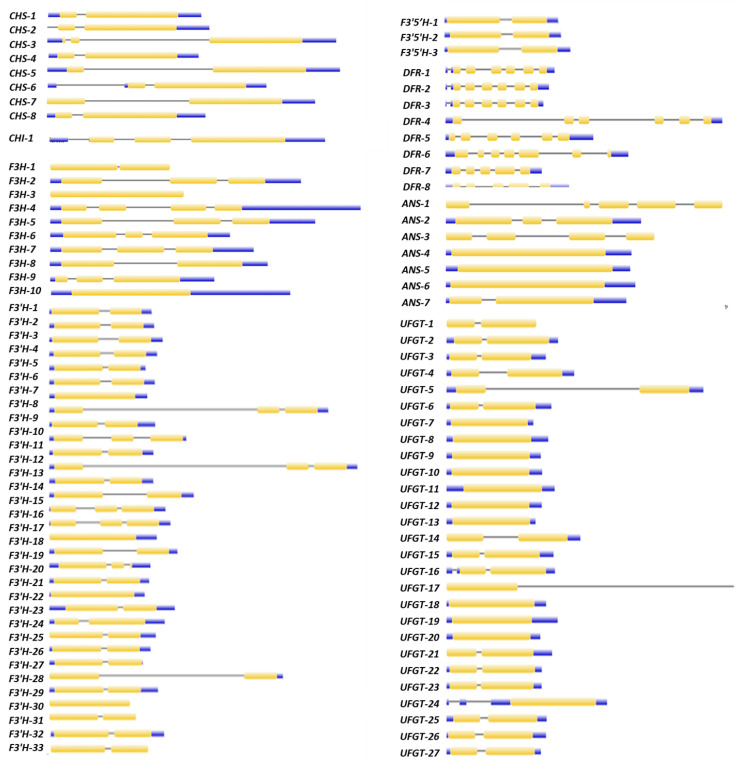
Exon–intron architecture of target genes. The gene structure was constructed by using the Gene Structure Display Server (GSDS). Exons are represented by yellow boxes and the introns are represented by the black lines.

The gene structure (Figure 5) and depicted motifs (Figure S1) of DEGs pertaining to the structural genes of the anthocyanin biosynthesis pathway were analyzed and represented in figure form. The gene structure analysis of eight CHS DEGs revealed that each DEG had 1–2 introns and a CDS. The motif analysis revealed that eight CHS DEGs had 5–10 motifs ranging in width from 8 to 50 in length. Motifs one, two and four were present in all eight DEGs; motifs three and five were present in seven DEGs; motifs six, seven and nine were present in six genes; and motifs eight and ten were present in five DEGs. The DEG CHI contained one intron and two CDS regions. DEG CHI contained ten motifs, according to the motif analysis. The number of F3H DEGs identified in wheat was 10 out of 101 putative genes. The F3H DEGs contained 3–10 motifs ranging in length from 15 to 50 amino acids. Of the 33 F3′H DEGs, 23 had two CDSs and one intron, six had three CDS and two introns, and four had one CDS and no intron. The predicted motifs ranged in length from 16 to 41 amino acids. Motifs 1–5 and 7 were present in all F3′H DEGs, while motifs 6 and 9–10 were present on 31 genes, and motif 8 was present on 28. The gene structure of the F3′5′H DEGs revealed that all three DEGs had two CDSs and one intron. Motifs 1–2, 4–5 and 9 were found among the three DEGs, while motifs 3,6,7–8 and 10 were found on two genes. According to the gene structure of DFR DEGs, five genes have six CDSs and five to six introns, one gene has seven CDSs and six introns, and two genes have five CDSs and four introns, the predicted motifs ranging in length from 8 to 50 amino acids. Motifs 1–6 and 8 were found in all eight DEGs, while motif 7 was found on seven genes and motif 9–10 on three. The gene structure of seven ANS DEGs revealed that three had one CDS but no intron, one had two introns and three CDSs, one had four CDSs and three introns, and one had five CDSs and four introns. ANS DEGs had motifs ranging in length from 13 to 50 amino acids. Motifs 1–2 and 8 were found on seven genes, motifs 3–4 and 10 on six genes, and motifs 5–7 and 9 on only three genes. The gene structure of 27 UFGT DEGs revealed that fifteen DEGs had two CDSs and one intron, ten DEGs had one CDS but no intron, one gene had one CDS and two introns, and one gene had one CDS and one intron. The motif specific to each biosynthetic gene has definite functions (Table S1).

### 2.6. Cis-Acting Regulatory Elements (CAREs)

**Figure 6:**
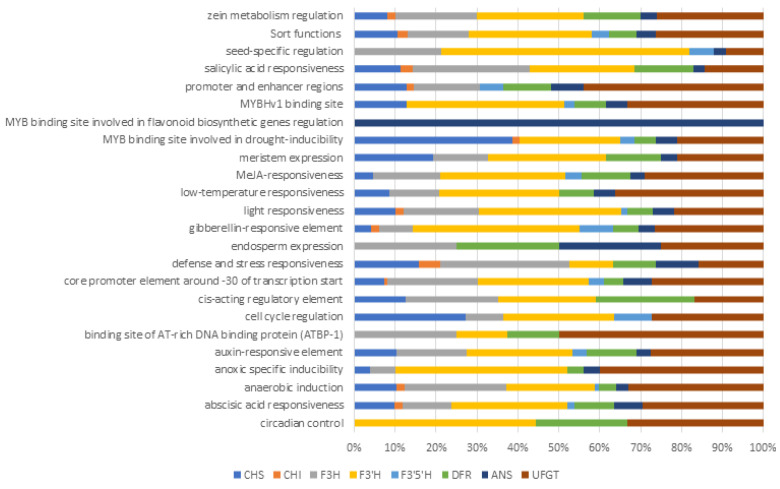
Most commonly occurring *cis*-acting regulatory elements in target genes.

Non-coding DNA components called CAREs; found in the promoter region of genes, they play a key role in gene regulatory networks and regulate gene expression. Additionally, they provide information on the physiological process and potential gene involvement. In all anthocyanin biosynthetic genes, most of the CAREs are involved in stress responses such as light, low temperature, salicylic acid, drought, anoxic, abscisic acid, etc., as well as the core promoter functions (Figure 6 and Table S3). *F3′5′H* and *UFGT* upstream region showed a large number of CAREs compared to other biosynthetic genes. Seed and endosperm-specific regulating CAREs were observed in later-stage biosynthetic genes, i.e., F3H, F3′H, F3′5′H, DFR, ANS and UFGT, respectively. Captivatingly, various MYB binding sites were observed in upstream regions of all biosynthetic genes, but the MYB binding site for flavonoid biosynthesis regulation is present only in the *ANS* upstream region.

### 2.7. Phylogenetic and Homolog Analysis

**Figure 7:**
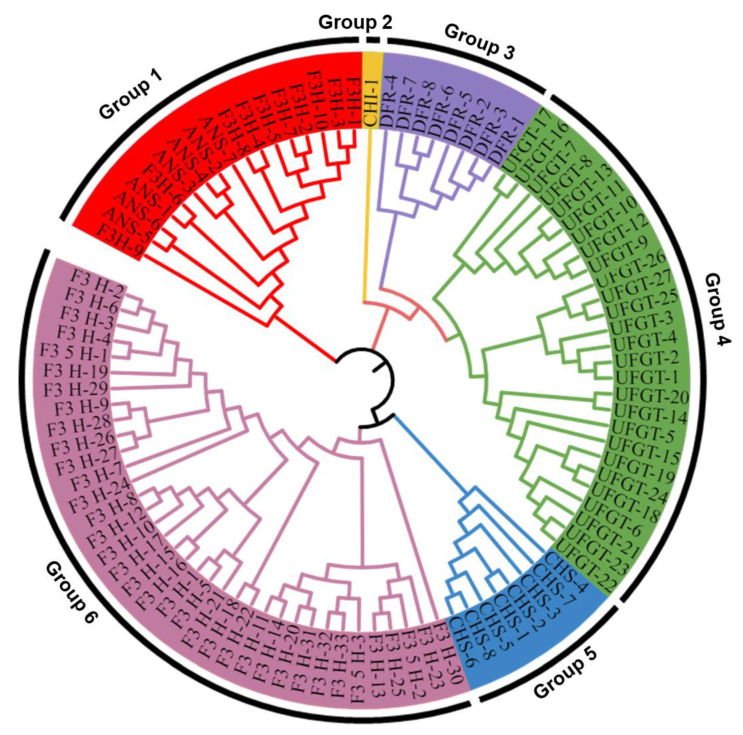
Phylogenetic tree showing the clustering of various target genes. Different colors depict the different gene groupings based on protein similarity. Blue represents CHS genes; purple represents DFR genes; pink represents the F3′H and F3′5′H genes; yellow represents the CHI gene; red represents the combined F3H and ANS genes; and green represents UFGT genes.

A phylogenetic tree of DEGs of each target gene was generated using multiple sequence alignment using a muscle algorithm (Figure 7). The genes pertaining to various target anthocyanin biosynthesis genes were clustered based on protein similarity. The putative CHS genes were grouped in group five, the putative DFR gene was clustered in group three, the putative F3′H and F3′5′H genes were grouped in group six, the putative CHI gene was grouped alone in group two, group one consisted of putative F3H gene, and, ANS genes and UFGT genes were clustered separately in group four. Interestingly, *F3H* and ANS isoforms were placed in one group, though functionally work apart. This perusal of the phylogeny tree indicated that the origin of anthocyanin biosynthetic genes in plants occurred randomly or that the genes play different functional roles in other pathways.

**Figure 8:**
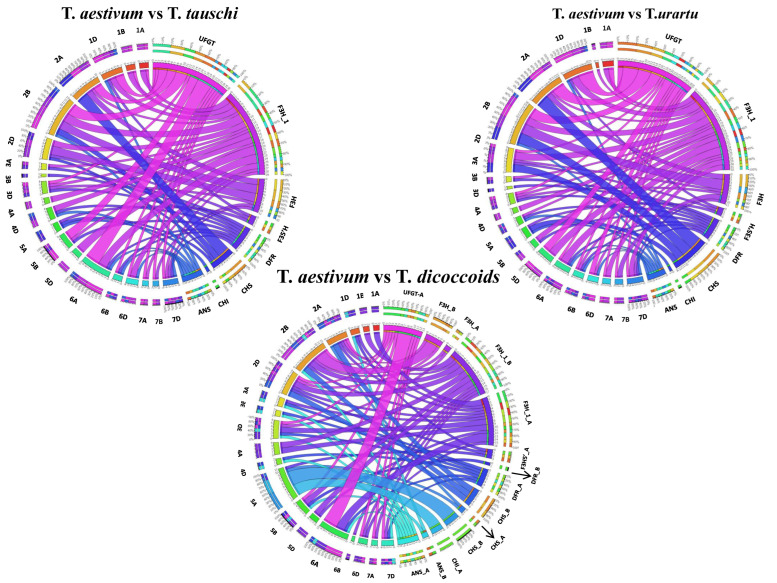
Syntenic relationship of target genes orthologous with their close relatives, *Ae. tauschii*, *T. urartu* and *T. dicoccoides*. Chromosomes represent the respective ancestral parent genome (*tauschii*/*urartu*/*dicoccoides*) and gene names represent the *T. aestivum* genome part.

We further tried understanding the syntenic link between wheat and its genome contributors because wheat evolved through hybridization. Chromosome-wise classification of orthologous anthocyanin biosynthetic genes in *Triticum urartu*, *Aegilopus tauchi,* and *T. dicoccoides* was depicted by the circular ideogram of Circos (Figure 8). The anthocyanin biosynthesis genes of *Ae*. *tauschii* (D-donor), *T*. *urartu* (A-donor) and *T*. *dicoccoides* (a tetraploid, AB genome) showed high levels of conservation as predicted. On *T*. *urartu* (A homoeologs) and *Ae*. *tauschii* (D homoeologs), it was shown that up to 60–80% of the anthocyanin biosynthetic genes of each chromosome were syntenic with their corresponding chromosomes; however, this percentage was higher in *T*. *dicoccoides* (Figure 8).

## 3. Discussion

Anthocyanins are health-promoting bioactive compounds synthesized via the phenylpropanoid pathway, a central pathway for synthesizing phenolics, stilbenes, flavonols, flavonoids and many more bioactive compounds. The anthocyanin biosynthesis pathway, which includes several structural, regulatory and transport genes, has been well characterized in *O. sativa*, *A. thaliana* and *Brassica* spp. [18,19,20]. Little is known about the anthocyanin biosynthesis pathway in wheat due to a lack of genome information. The recently fully sequenced wheat genome has enabled researchers worldwide to gain a deep insight into the wheat genome’s hidden molecular and structural aspects [21]. Researchers used to focus on developing wheat varieties with high yields, protein and resistance to biotic and abiotic stresses [22,23] Nevertheless, wheat varieties with high flavonoid content have recently become popular because of their health benefits [24,25,26,27,28]. Anthocyanin-rich color wheat varieties have been introduced with a vision of better health for a population. This study involved the identification of the putative structural genes of wheat anthocyanin biosynthesis pathway in black, blue, purple and white wheat and their comparative expression analysis by in silico as well as RNA-Seq approaches. Eight targeted structural genes, including early biosynthesis genes (CHS, CHI, F3H, F3′H and F3′5′H) and late biosynthesis genes (DFR, ANS and UFGT) [29], were characterized in this study. The F3′H genes were identified in the maximum number, followed by UFGT genes. CHI genes were identified in the least number. Such a large number of target genes identified in wheat might be due to the big and complex genome size. During evolution, wheat has undergone many genome replication events, which gave rise to allohexaploid wheat with three subgenomes, A, B and D [30]. Therefore, wheat may contain multiple genes, known as homeologs, which have varying localization within the genome [31]. One of the studied genes, CHS, is an omnipresent enzyme in many plant species, including gymnosperms and angiosperms, and it initiates flavonoid biosynthesis. It is a plant-specific polyketide synthase reported in plants such as poplar, Arabidopsis, snapdragon, morning glory, flax, maize and rice [32,33,34,35]. The identified “Chal_sti_synt_N” domain and “Chal_sti_synt_C” in putative CHS genes in wheat are consistent with other plants such as maize, rice and poplar [18,33,36]. The exon–intron organization of putative CHS in wheat was consistent with other plant species’ findings [37,38]. The NCBI CDD search confirmed the presence of conserved catalytic triad Cys-His-Asn at the active site [39], which helps in the multiple decarboxylation and condensation reactions. Another studied gene, CHI, exists as a multigene family and catalyzes the formation of (2S)-5-hydroxy flavanone from 6′-hydroxy chalcone (naringenin chalcone). The domain analysis of *CHI* depicted the presence of the “chalcone domain,” and the gene structure showed the presence of three introns. Similar findings were made in red sage [38]. The next studied gene, F3′H, plays a role in the 3-hydroxylation of flavanone to form dihydroflavanol [19]. It is a member of the 2OG-Fe (II) oxygenase superfamily, a class of iron-containing non-heme oxygenases localized in the cell’s cytosol [40]. The putatively identified F3′H gene has a characteristic 2_ODD domain supported by the Pfam and NCBI CDD domain search results. F3′H and F3′5′H genes are members of the cytochrome P450 monooxygenase superfamily and catalyze the hydroxylation step to impart purple–blue pigments [41]. Phylogeny analysis grouped these two genes under a single group, indicating that they might come from a common ancestor and share a common Pfam domain. The next gene, DFR, is another equally important enzyme of the nicotinamide adenine dinucleotide dependent epimerase family/dehydratase family. The DFR enzyme has a wide range of substrate specificity, which controls various flower colors and seed coat pigmentation [42]. All the putative DFR proteins possessed the conserved epimerase domain and the Ser-Tyr-Lys catalytic active site triad as detected by NCBI-CDD. The crystal structure of the DFR protein from grapes has confirmed the presence of a similar catalytic triad. An epimerase domain specific to the DFR protein has been confirmed in red sage and other plant species [38,43]. Another studied biosynthetic gene, ANS, plays a key role in anthocyanin biosynthesis, which converts leucoanthocyanidin to anthocyanidin [40]. The Pfam domain analysis suggested that the ANS gene belongs to the 2-oxoglutarate dependent dioxygenase (2- ODD) superfamily and is composed of a DIOX_N domain and 2OGFeII_Oxy terminal domain. Results were consistent with the previously reported studies in Chinese yew [44]. The next studied gene, UFGT, is the last in the anthocyanin pathway and has glycosyltransferase activity. We identified 242 UFGT genes in wheat. This number is higher than the 179 putative wheat UFGT genes found earlier [45] as we used the recently fully sequenced wheat genome [21]. Many putative UFGT genes have also been identified in soybean [46,47]. Likewise, 47 *UFGT*s were identified in maize [48]. Domain analysis indicated the presence of the UDP-glycosyltransferase domain, as reported previously [45].

Many colored plants have been studied for the structural genes in the anthocyanin production pathway [49,50]. Accordingly, the anthocyanin biosynthesis pathway in colored wheat was better comprehended in the current study due to the transcriptome profiling and in silico studies. Despite the number of putative gene isoforms in the wheat genome, only a tiny fraction of each gene showed differential expression between anthocyanin biofortified wheat and white wheat. This might be due to the polyploid nature of wheat, where gene duplication often occurs [51], and the genes might be expressed differentially in a tissue-specific manner or functional diversification. Some evolution events such as duplication could extend the members of plant gene families and mutations in the regulatory regions and coding sites could alter the expression patterns and function of new members [52,53]. As we also observed in the Circos figure (Figure 7) that some of the colored wheat anthocyanin biosynthetic genes have synteny with their progenitors, their number grew through gene complementation during evolution and polyploidization to confer adaptive plasticity [30].

Glagoleva et al. [54] reported that only eight isoforms of *CHS* sustained their original structure and activity. We observed a higher expression of *CHS* isoforms, specifically the 2A and 2D isoforms, in purple wheat compared to blue and black wheat. A similar homeolog of CHS genes was observed in the 2A and 2D chromosomes [54]. The CHS family is not only known for anthocyanin synthesis but also imparts multiple functions and is thus expressed more in the purple pericarp, which is an outer layer associated with abiotic/biotic stress responses [55,56,57].

The CHI gene has been localized to the homoeologous group five chromosome by genetic mapping studies [58]. Interestingly, in our study, there was only one differentially expressed CHI gene, which was found on the 5A chromosome. The *F3H* alleles have been found to be located on 2A, 2B and 2D chromosomes by genetic mapping [59]. Our study found the highest number of differentially expressed F3H genes on homoeologous group two chromosomes, and the highly expressed IDs TraesCS2B02G521500, TraesCS2D02G493400 and TraesCS2A02G493500 localized on group two chromosomes formed a distinct clad, indicating their close structure relation in phylogenetic analysis. It was highly expressed in purple and white wheat, indicating its multiple involvements, such as flavonoids, anthocyanins and carotenoids. On the other hand, black and blue wheat exhibit higher DEGs related to putative *F3′5′H* (TraesCS1D02G403300) than purple wheat. It has been determined that the primary enzyme responsible for the synthesis of delphinidin is *F3′5′H* [59]. F3H, F3′H and F3′5′H are the main enzymes accounting for the diversification of anthocyanins by governing their B-ring hydroxylation pattern and, ultimately, their color [7]. According to Sharma et al. [60], black and blue wheat show a high concentration of delphinidin-based anthocyanins, which impart a blue hue to the plants [61]. Various isoforms of all anthocyanin biosynthesis genes also showed expression in white wheat, indicating that visual color development is a complex process and is not solely dependent on anthocyanin biosynthesis structural genes.

Localization of *DFR* and *ANS* have been reported on homoeologous group three and group six chromosomes, respectively [59]. The highest number of differentially expressing DFR genes were observed in homoeologous group three in our study, but their expression was higher in white wheat, indicating multiple involvements. The highest number of differentially expressing ANS genes was observed in homoeologous group six in our study. Thus, our data complement the genetic mapping studies. However, *ANS* differentially expressed isoforms showed variable expression patterns in purple, blue and black wheat. *ANS* is known to be majorly involved in color development [44,62,63,64,65]. The blue wheat used in this study is a substitution line with the replacement of the 4D chromosome of wheat with 4E of *Agropyron elongatum* {4E(4D)}. However, none of the differentially expressed biosynthetic genes belonged to chromosome 4D in purple, blue or black wheat, indicating that some genes on 4E may be regulating genes acting on biosynthetic genes on different chromosomes. Further, the involvement of 4D with the anthocyanin biosynthetic pathway is very little, and thus its replacement with 4E might have provided the key missing gene in white wheat and led to the development of the blue aleurone color.

Numerous researchers reported that anthocyanins play essential roles in response to abiotic and biotic stresses for plants [66,67]. Moreover, different abiotic and biotic stress-responsive regulatory elements were observed upstream for these anthocyanin biosynthesis genes. These regulatory elements might be responsible for enhanced anthocyanin biosynthesis in response to light, low temperature and other abiotic stresses [58,68]. It has also been observed that only the *ANS* upstream region showed an “MYB binding site” specific for flavonoid biosynthesis regulation. It has been functionally validated in petunia (PlantCARE database). It further supports the hypothesis that *ANS* may be one of the significant genes involved in anthocyanins biosynthesis. The association of *ANS* with color development has been proven by different loss of function studies including insertion mutation in *ANS* in pomegranate [62], loss of *ANS* in endophytic fungi *Salvia miltorrhiza* [65], the mutation in *ANS* in sweet basil [64] and raspberry [63], and the methylation pattern in peach [69].

Four of the eight studied genes showed endosperm expression-specific promoter binding sites. As colored wheat develops anthocyanins in the seed coat, and its endosperm development is desirable, expressing unexpressed genes can assist specific sites rather than the whole set of genes [70]. This research shows that wheat’s anthocyanin biosynthesis pathway genes and their regulatory binding sites need more exploration and functional validation.

## 4. Materials and Methods

### 4.1. Genome-Wide Identification and Distribution of Anthocyanin Biosynthetic Genes in Wheat

To identify the key structural genes of the anthocyanin biosynthesis pathway in colored wheat, the corresponding amino acid sequences of CHS, CHI, F3H, F3′H, F3′5′H, DFR, ANS and UFGT from the *O. sativa* and *A. thaliana* (Ensemble Plants) were downloaded. Blastp search was performed against the wheat protein database downloaded from the ensemble plants (https://plants.ensembl.org/Triticum_aestivum/Info/Index, accessed on 20 July 2021) with an *e*-value cutoff of 0.00001 and bit-score > 100.

Genes’ distribution was analyzed by mapping them across the wheat chromosomes, and a corresponding pictorial representation was generated using Map chart software (version 2.32). The Ensemble Plants wheat database was used to acquire the necessary gene location information (http://archive.plants.ensembl.org/Triticum_aestivum/Info/Index, accessed on 20 July 2021).

### 4.2. Comparative Transcriptome Data Analysis of Anthocyanin Biosynthesis Related Genes in Color Wheat

#### 4.2.1. Plant Material

High anthocyanin content color wheat advanced lines (black, blue and purple) [71,72,73] and common amber wheat (cv. PBW621) lines were grown at the National Agri-Food Biotechnology Institute (NABI) in Mohali, India (30.7046° N, 76.7179° E). Ten spikes with developing seeds (corresponds to 28 days after anthesis (DAA) or Zadoks scale 85 (soft dough)) were harvested and stored at −80 °C. The same were used for RNA isolation.

#### 4.2.2. RNA Isolation and RNA-Seq Analysis

RNA from three biological replicates was isolated using the Sigma Spectrum plant’s total RNA kit, followed by mRNA extraction, library preparation and paired-end sequencing using the illumina platform. The raw reads obtained after sequencing were checked for adapter contamination and low-quality base pairs (q < 20) using FastQC (https://www.bioinformatics.babraham.ac.uk/projects/fastqc/, accessed on 14 January 2023) and trimmed using bbuk from the BBtools package (https://jgi.doe.gov/data-andtools/bbtools/bb-tools-user-guide/bbduk-guide/, accessed on 14 January 2023). Annotated wheat transcripts were retrieved from Ensemble Plants (Ensemble release 47—April 2020), and the high-quality reads were mapped to them using salmon (PMC5600148). The resulting transcript level counts were collapsed into gene-level counts using tximport [74] and transferred to DESeq2 [75] for differential gene expression analysis. The resulting *p*-values were corrected for multiple hypothesis testing using the Benjamini and Hochberg method implemented inside the DESeq2 package. An FDR < 0.05 was considered significant, and selected genes were used for downstream processing. Alternatively, the CLC workbench was also employed for RNA seq data analysis. The differentially expressed genes pertaining to the key structural genes of the anthocyanin biosynthesis pathway were examined for gene ontology and motif prediction.

#### 4.2.3. Characterization of Identified Anthocyanin Biosynthetic Genes

Subcellular localization of the key structural genes was determined in silico by using WoLF PSORT and Plant-mPLoc [76,77], prediction programs hosted online. Isoelectric point and molecular weight were determined using an isoelectric point calculator (isoelectric.org, accessed on 14 January 2023).

Conserved domains present in the key structural genes were identified using NCBI CDD (https://www.ncbi.nlm.nih.gov/Structure/cdd/cdd.html, accessed on 14 January 2023) and HMMscan (https://www.ebi.ac.uk/Tools/hmmer/search/hmmscan, accessed on 14 January 2023).

#### 4.2.4. Gene Motif, and Cis-Acting Regulatory Elements (CAREs) Identification

For the identification of motifs, the Multiple Expectation Maximization for Motif Elicitation (MEME) program version 4.11.4 (http://meme-suite.org, accessed on 14 January 2023) was used [78]. The identified motif was searched using the SMART database to confirm the presence of gene-specific domains. The following parameters were specified to run the MEME: maximum motif width between 6 and 50 and a maximum number of motifs of 10.

The PlantCARE database of plant cis-acting regulatory elements and a tool for in silico analysis of promoter sequences (https://bioinformatics.psb.ugent.be/webtools/plantcare/html/, accessed on 14 January 2023) were used to identify the CAREs or in silico promoter sequences. A total of 1500 bp upstream sequences of the anthocyanin biosynthetic genes, as mentioned earlier, were obtained from Ensemble Plants and examined using the PlantCARE database. From each isoform of the CHS, CHI, F3′H, F3′5′H, DFR, ANS and UFGT genes, CARE motifs were collected, filtered as function-specific and represented in figure form.

#### 4.2.5. Phylogenetic and Homology Analysis

Multiple sequence alignment was performed using the muscle algorithm [79] on protein sequences corresponding to the DEGs of key structural genes, and a phylogenetic tree was built using the Neighbor-Joining (NJ) technique in MEGA X software [80].

Ensemble Plants’ CDS sequences for *T*. *urartu* (ftp://ftp.ensemblgenomes.org/pub/plants/release-42/fasta/triticum_urartu, accessed on 14 January 2023), *Ae. tauschii* (ftp://ftp.ensemblgenomes.org/pub/release-42/plants/fasta/aegilops_tauschii/cds, accessed on 14 January 2023) and *T*. *dicoccoides* (ftp://ftp.ensemblgenomes.org/pub/release-42/plants/fasta/triticum_dicoccoides/cds/, accessed on 14 January 2023), which make up the A, D and AB genomes, respectively, were downloaded in order to identify orthologous genes in close relatives of *T*. *aestivum*. The synteny relationship within the different genome donators was performed using Circo’s online server (http://mkweb.bcgsc.ca/tableviewer/visualize/, accessed on 14 January 2023). A 150-bit-score cutoff and an *e*-value cutoff of 1E-10 were used to filter the findings. The top hits were considered orthologs in that species.

#### 4.2.6. qRT-PCR Validation of Anthocyanin Biosynthetic Genes during Seed Development

Primer3 (https://www.primer3plus.com/, accessed on 14 January 2023) was used to create genome-specific primers for putative anthocyanin biosynthesis genes (Table S4: list of primers) and wheat ADP-Ribosylation Factor (ARF) and Glyceraldehyde 3-phosphate dehydrogenase (GAPDH) were taken as an internal control. The 7500 Fast Real-Time PCR System was used to conduct qRT-PCR analysis on three biological replicates and their corresponding three technical duplicates using 1:10 diluted cDNA and Fast SYBR Green master mix. The script DNA clear cDNA synthesis kit (Bio-Rad CFX96, USA) was used for cDNA preparation. For the qRT-PCR study, RNAs were isolated as depicted previously from 28DAA. The default cycle parameters (Bio-Rad, USA Amplifier) for amplification were set, and fold change values based on 2^-ΔΔCT^ values were used to plot graphs using ARF as a stable internal control. Graph Pad Prism (version 5) was used for their significance level with a cutoff of *p*-value > 0.05.

## 5. Conclusions

The present study systematically identified and characterized the anthocyanin biosynthesis genes at the wheat genome level and characterized them in silico. This research enables a better understanding of anthocyanin biosynthetic genes in wheat for its nutritional enhancement. Colored wheat is a promising option for the increasing demand for nutrition-dense foods worldwide. Colored wheat will also be a good alternative as an abiotic and biotic stress-tolerant crop given climate change. We still need to explore and know more about anthocyanin biosynthetic genes in wheat to receive the best benefits and assist in developing anthocyanin in the highly consumed part of the seed, i.e., the endosperm. These putative structural genes are also implicated in the reactions to light, drought, low temperature and other defense mechanisms. The results of this study will advance the knowledge of anthocyanin synthesis at the gene expression level and set the stage for future modifications to wheat cultivars and the development of novel cultivars with enhanced nutritional value.

## Figures and Tables

**Table 1:** Detailed information of putatively identified structural genes of anthocyanin biosynthesis pathway in wheat.

S.No.	Gene Name	Abbreviation	Gene Superfamily	Number of Genes in *Triticum aestivum* (% Identity)
**1**	Chalcone synthase	*CHS*	Polyketide synthase enzymes (PKS)	95 (37–92)
**2**	Chalcone isomerase	*CHI*	Isomerases	7 (50–61)
**3**	Flavanone hydroxylase	*F3H*	2OG-Fe (II) oxygenase superfamily	101 (37–58)
**4**	Flavonoid 3′-hydroxylase	*F3*′*H*	P450-dependent monooxygenase (P450)	491 (37–81)
**5**	Flavonoid 3′,5′-hydroxylase	*F3*′*5*′*H*		51 (39–40)
**6**	Dihydroflavanol reductase	*DFR*	NAD dependent epimerase/dehydratase	133 (37–66)
**7**	Anthocyanidin synthase	*ANS*	2OG-Fe (II) oxygenase superfamily	73 (27–68)
**8**	UDP glycosyltransferases	*UGFT*	UDP-glucosyl transferases	242 (38–76)

**Table 2:** Physiochemical properties and identified domains in the structural genes of anthocyanin biosynthesis pathway in wheat.

Gene	Subcellular Localization	Predicted Molecular Mass KD (Min–Max)	Isoelectric Point (Min–Max)	Domain
*CHS*	Cytoplasm	23.6–52.04	5.06–9.21	Chal_sti_synt
*CHI*	Cytoplasm	23.54–24.18	4.54–5.09	Chalcone
*F3H*	Cytoplasm	30.1–48.13	4.85–8.04	2-Oxoglutatate dependent dioxygense
*F3′H*	Chloroplast	21.20–63.69	5.76–9.95	Cytochrome p450(P450-dependent monooxygenase)
*F3′5′H*	Chloroplast	39.27–66.59	5.63–9.91	Cytochrome p450(P450-dependent monooxygenase)
*DFR*	Cytoplasm	16.06–46.68	4.74–9.02	Epimerase
*ANS*	Cytoplasm	29.45–47.62	4.61–8.95	2OG-Fe (II) oxygenase superfamily
*UFGT*	Chloroplast	28.05–76.67	4.60–8.41	Glycosyltransferase_GTB-type superfamily

## Data Availability

All original data and images will be provided upon request by the corresponding author.

## Supplementary Materials

The following supporting information can be downloaded at https://www.mdpi.com/article/10.3390/genes14040809/s1, Figure S1: motifs depicted in target genes. The conserved motifs of various structural genes were found using MEME. The conserved motifs are represented by different colored boxes and assigned numbers 1–10. The length of the boxes signifies motif length. Table S1: analyzed motifs of anthocyanin biosynthetic genes. Table S2: simple representation of Traes IDs along with expression values. Table S3: distribution of cis-regulatory element of all anthocyanin biosynthetic genes isoforms. Table S4: gene specific primer list used for qRT-PCR.
