# Supplementary material for: Genome–Transcriptome Transition Approaches to Characterize Anthocyanin Biosynthesis Pathway Genes in Blue, Black and Purple Wheat
Source: Genes (Basel). 2023 Mar 27;14(4):809. doi: 10.3390/genes14040809 (PMC10137985; doi:10.3390/genes14040809)
Supplement: Supplementary file 1 [file genes-14-00809-s001.zip › Figure S1 & Table S1.pdf]

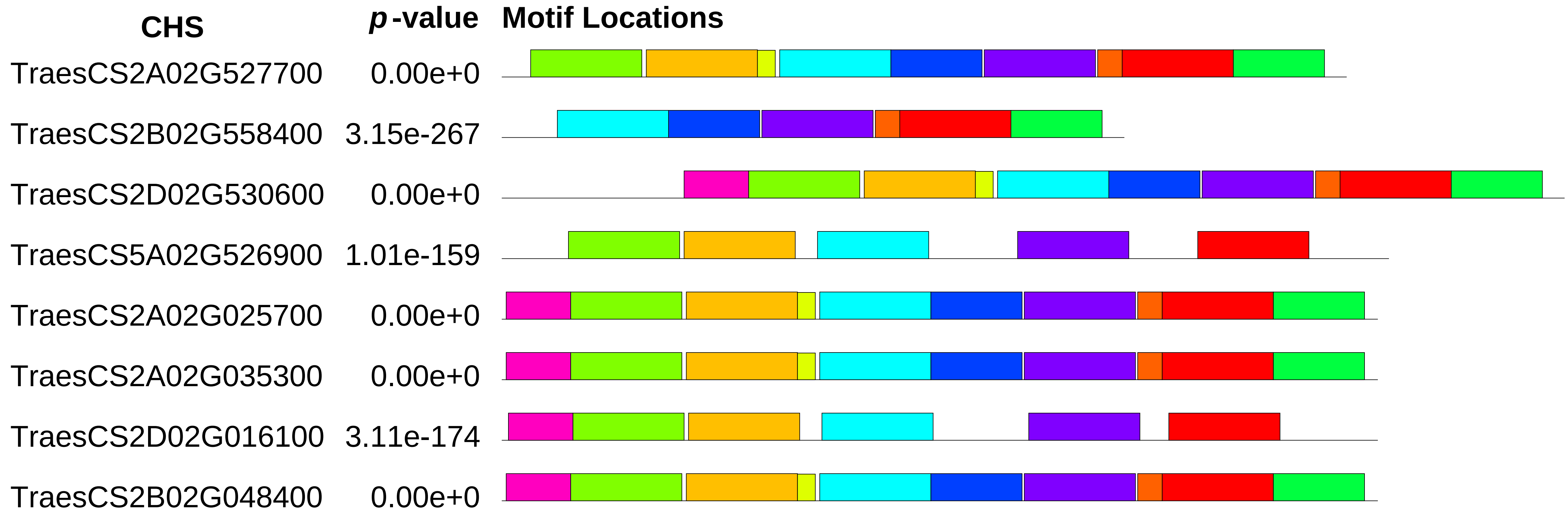

Motif	Symbol	Motif Consensus
1.		WNSVFWIAHPGGPAILDMVEAKVNLNKERMRATRHVLSEYGNMSSACVLF
2.		DYQLTKMLGLRPSVKRLMMYQQGCFAGGTVLRLAKDLAENNRGARVLVVC
3.		NCVYQADYPDYYFKITKSDHMADLKEKFKRMCDKSQIRKRYMHLTEEILQ
4.		ERPLFQLVSASQTILPDSEGAIDGHLREVGLTFHLLKDVPGLISKNIERA
5.		PNMCAYMAPSLDARQDIVVVEVPKLGKAAAQKAIKEWGQPRSKITHLVFC
6.		IMDEMRKRSAEDGHATTGEGMDWGVLFGFGPGLTVETVVLH
7.		SEITAVTFRGPHESHLDSLVGQALFGDGAAAVIIGADPDES
8.		ATMTVEEVRKAQRAEGPATVLAIGTATPA
9.		EDAFKPLGIDD
10.		TTSGVDMP

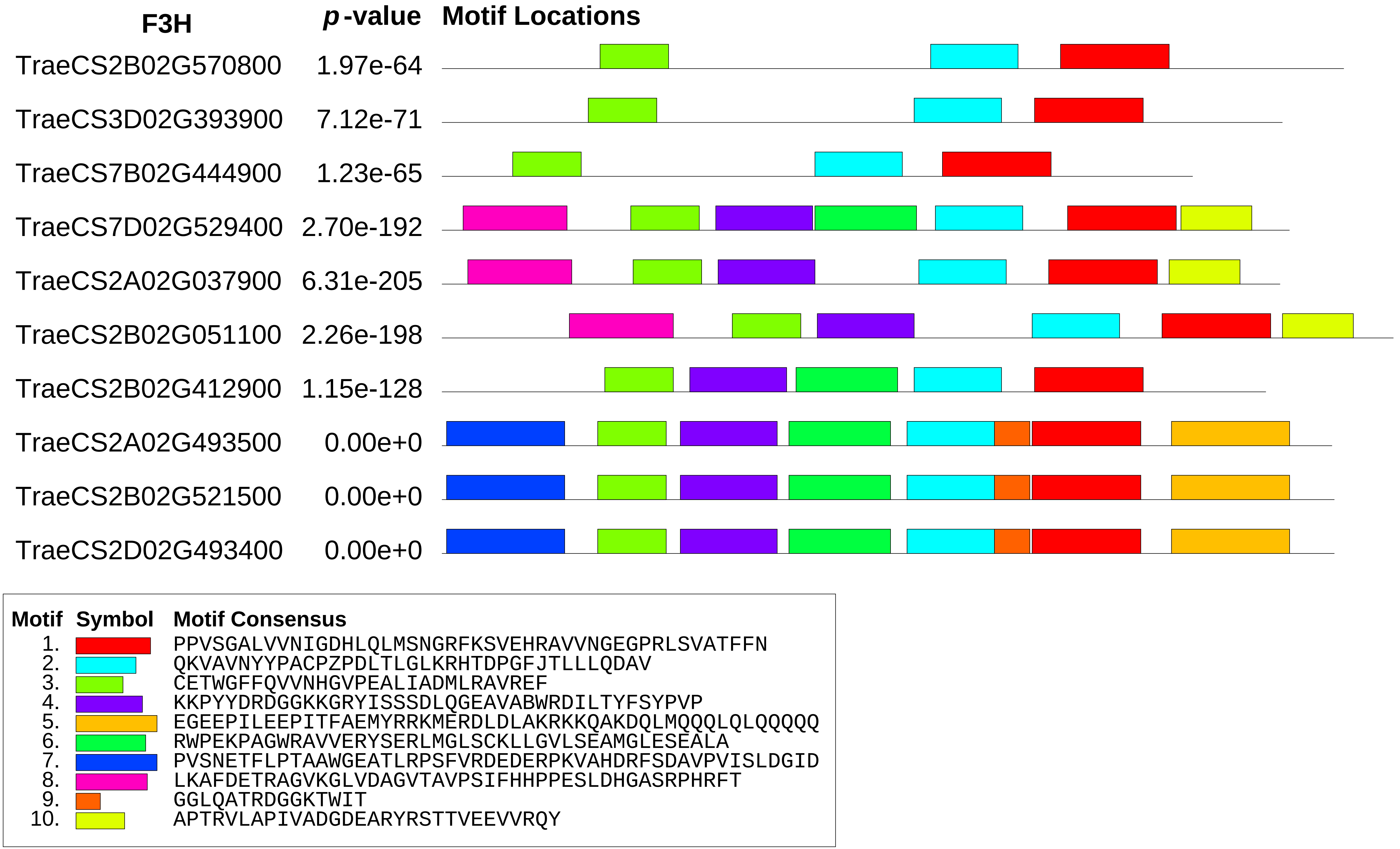
F3H
p-value
Motif Locations
TraeCS2B02G570800 1.97e-64
TraeCS3D02G393900 7.12e-71
TraeCS7B02G444900 1.23e-65
TraeCS7D02G529400 2.70e-192
TraeCS2A02G037900 6.31e-205
TraeCS2B02G051100 2.26e-198
TraeCS2B02G412900 1.15e-128
TraeCS2A02G493500 0.00e+0
TraeCS2B02G521500 0.00e+0
TraeCS2D02G493400 0.00e+0
Motif Symbol Motif Consensus
1. PPVSGALVVNIGDHLQLMSNGRFKSVEHRAVVNGEGPRLSVATFFN
2. QKVAVNYYPACPZPDLTLGLKRHTDPGFJTLLLQDAV
3. CETWGFFQVVNHGVPEALIADMLRAVREF
4. KKPYYDRDGGKKGRYISSSDLQGEAVABWRDILTYFSYPVP
5. EGEEPILEEPITFAEMYRRKMERDLDLAKRKKQAKDQLMQQQLQLQQQQQ
6. RWPEKPAGWRAVVERYSERLMGLSCKLLGVLSEAMGLESEALA
7. PVSNETFLPTAAWGEATLRPSFVRDEDERPKVAHDRFSDAVPVISLDGID
8. LKAFDETRAGVKGLVDAGVTAVPSIFHHPPESLDHGASRPHRFT
9. GGLQATRDGGKTWIT
10. APTRVLAPIVADGDEARYRSTTVEEVVRQY

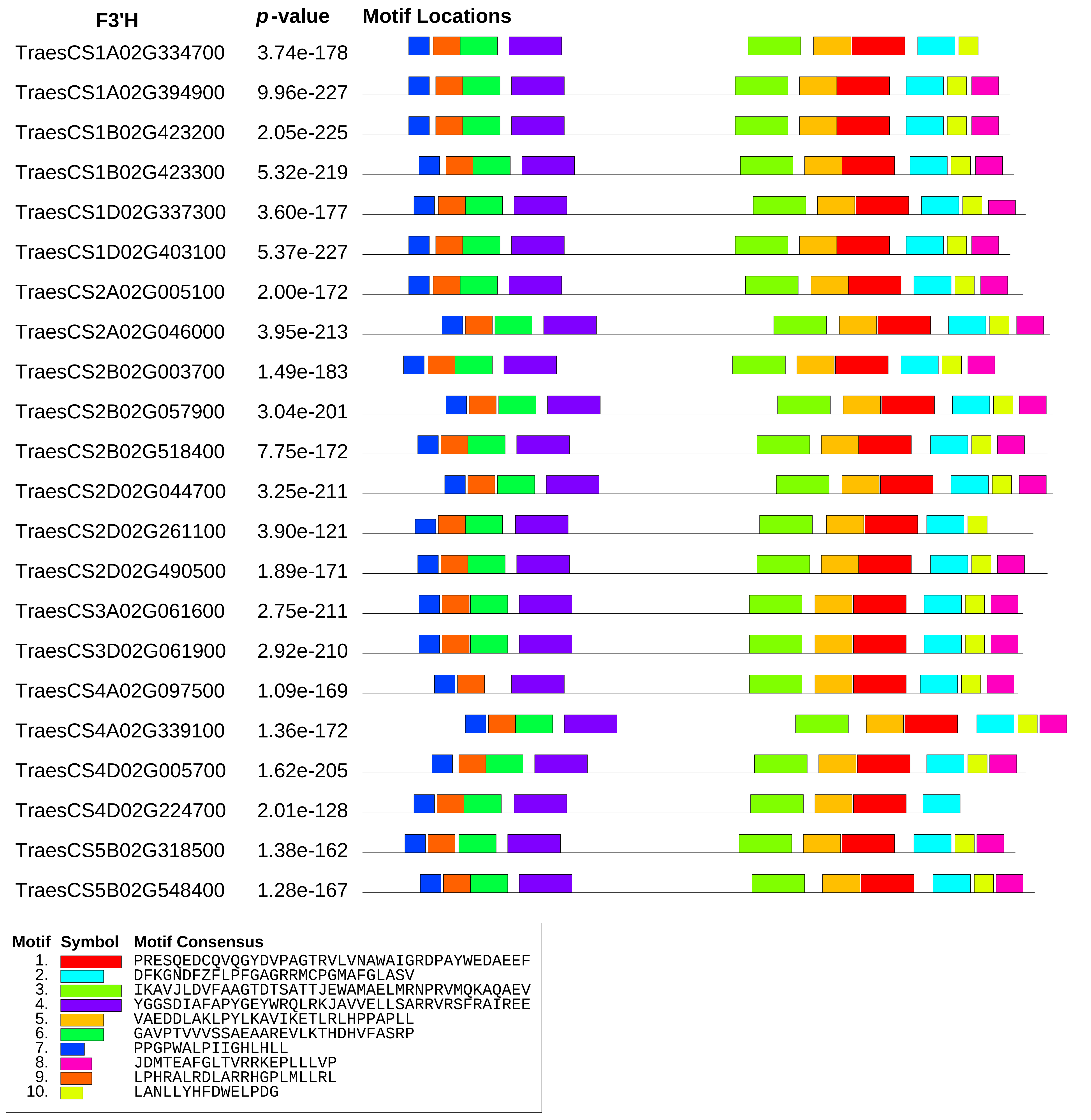
F3'H
p-value
Motif Locations
TraesCS1A02G334700 3.74e-178
TraesCS1A02G394900 9.96e-227
TraesCS1B02G423200 2.05e-225
TraesCS1B02G423300 5.32e-219
TraesCS1D02G337300 3.60e-177
TraesCS1D02G403100 5.37e-227
TraesCS2A02G005100 2.00e-172
TraesCS2A02G046000 3.95e-213
TraesCS2B02G003700 1.49e-183
TraesCS2B02G057900 3.04e-201
TraesCS2B02G518400 7.75e-172
TraesCS2D02G044700 3.25e-211
TraesCS2D02G261100 3.90e-121
TraesCS2D02G490500 1.89e-171
TraesCS3A02G061600 2.75e-211
TraesCS3D02G061900 2.92e-210
TraesCS4A02G097500 1.09e-169
TraesCS4A02G339100 1.36e-172
TraesCS4D02G005700 1.62e-205
TraesCS4D02G224700 2.01e-128
TraesCS5B02G318500 1.38e-162
TraesCS5B02G548400 1.28e-167
Motif Symbol Motif Consensus
1. PRESQEDCQVQGYDVPAGTRVLVNAWAIGRDPAYWEDAEEF
2. DFKGNDFZFLPFGAGRRMCPGMAFGLASV
3. IKAVJLDVFAAGTDTSATTJEWAMAELMRNPRVMQKAQAEV
4. YGGSDIAFAPYGEYWRQLRKJAVVELLSARRVRSFRAIREE
5. VAEDDLAKLPYLKAVIKETLRLHPPAPLL
6. GAVPTVVVSSAEAAREVLKTHDHVFASRP
7. PPGPWALPIIGHLHLL
8. JDMTEAFGLTVRRKEPLLLVP
9. LPHRALRDLARRHGPLMLLRL
10. LANLLYHFDWELPDG

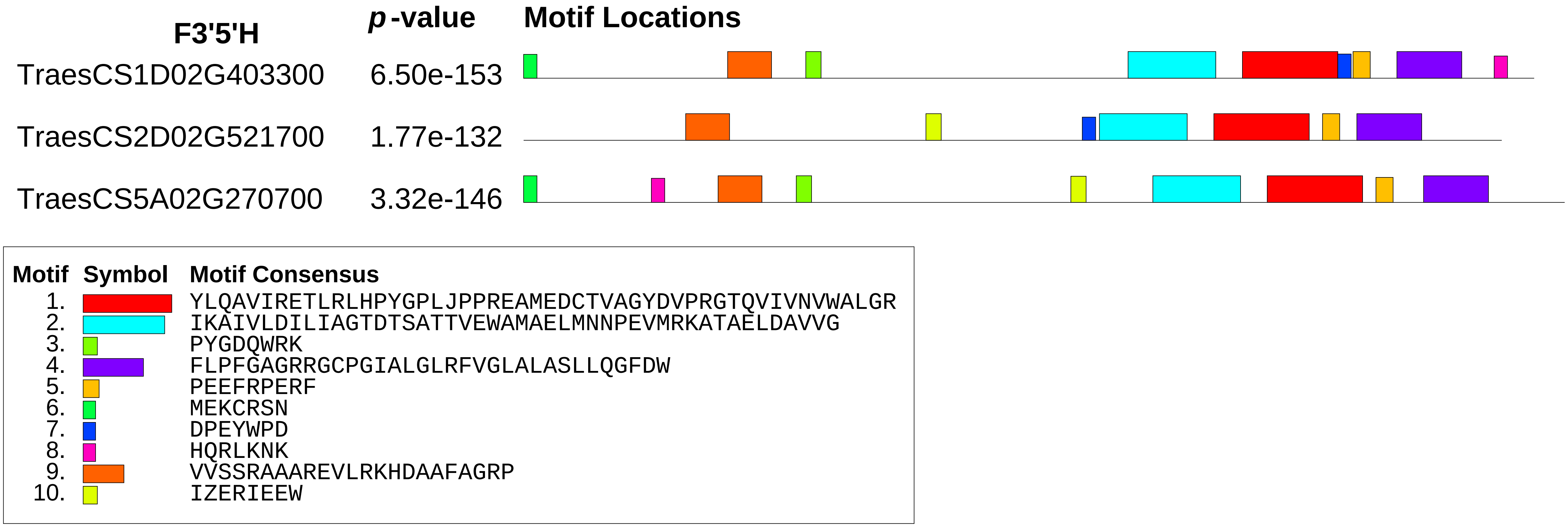
F3'5'H
p-value
Motif Locations
TraesCS1D02G403300
6.50e-153
TraesCS2D02G521700
1.77e-132
TraesCS5A02G270700
3.32e-146
Motif
Symbol
Motif Consensus
1. YLQAVIRETLRLHPYGPLJPPREAMEDCTVAGYDVPRGTQVIVNVWALGR
2. IKAIVLDILIAGTDTSATTVEWAMAELMNNPEVMRKATAELDAVVG
3. PYGDQWRK
4. FLPFGAGRRGCPGIALGLRFVGLALASLLQGFDW
5. PEEFRPERF
6. MEKCRSN
7. DPEYWPD
8. HQRLKNK
9. VVSSRAAAREVLRKHDAAFAGRP
10. IZERIEEW

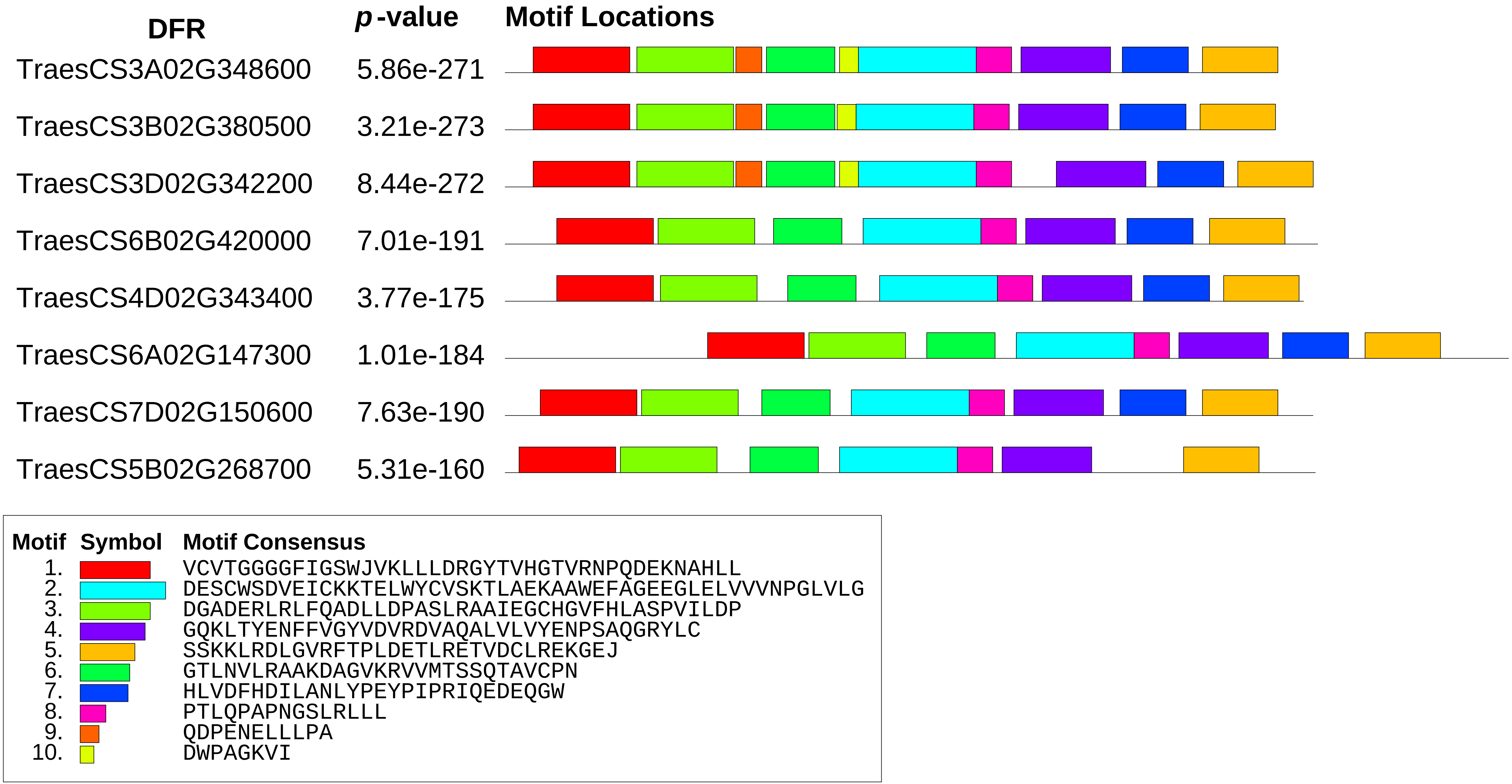
DFR
p-value
Motif Locations
TraesCS3A02G348600 5.86e-271
TraesCS3B02G380500 3.21e-273
TraesCS3D02G342200 8.44e-272
TraesCS6B02G420000 7.01e-191
TraesCS4D02G343400 3.77e-175
TraesCS6A02G147300 1.01e-184
TraesCS7D02G150600 7.63e-190
TraesCS5B02G268700 5.31e-160
Motif Symbol Motif Consensus
1. VCVTGGGGFIGSWJVKLLLDRGYTVHGTVRNPQDEKNAHLL
2. DESCWSDVEICKKTELWYCVSKTLAEKAAWEFAGEEGLELVVVNPGLVLG
3. DGADERLRLFQADLLDPASLRAAIEGCHGVFHLASPVILDP
4. GQKLTYENFFVGYVDVRDVAQALVLVYENPSAQGRYLC
5. SSKKLRDLGVRFTPLDETLRETVDCLREKGEJ
6. GTLNVLRAAKDAGVKRVVMTSSQTAVCPN
7. HLVDFHDILANLYPEYPIPRIQEDEQGW
8. PTLQPAPNGSLRLLL
9. QDPENELLLPA
10. DWPAGKVI

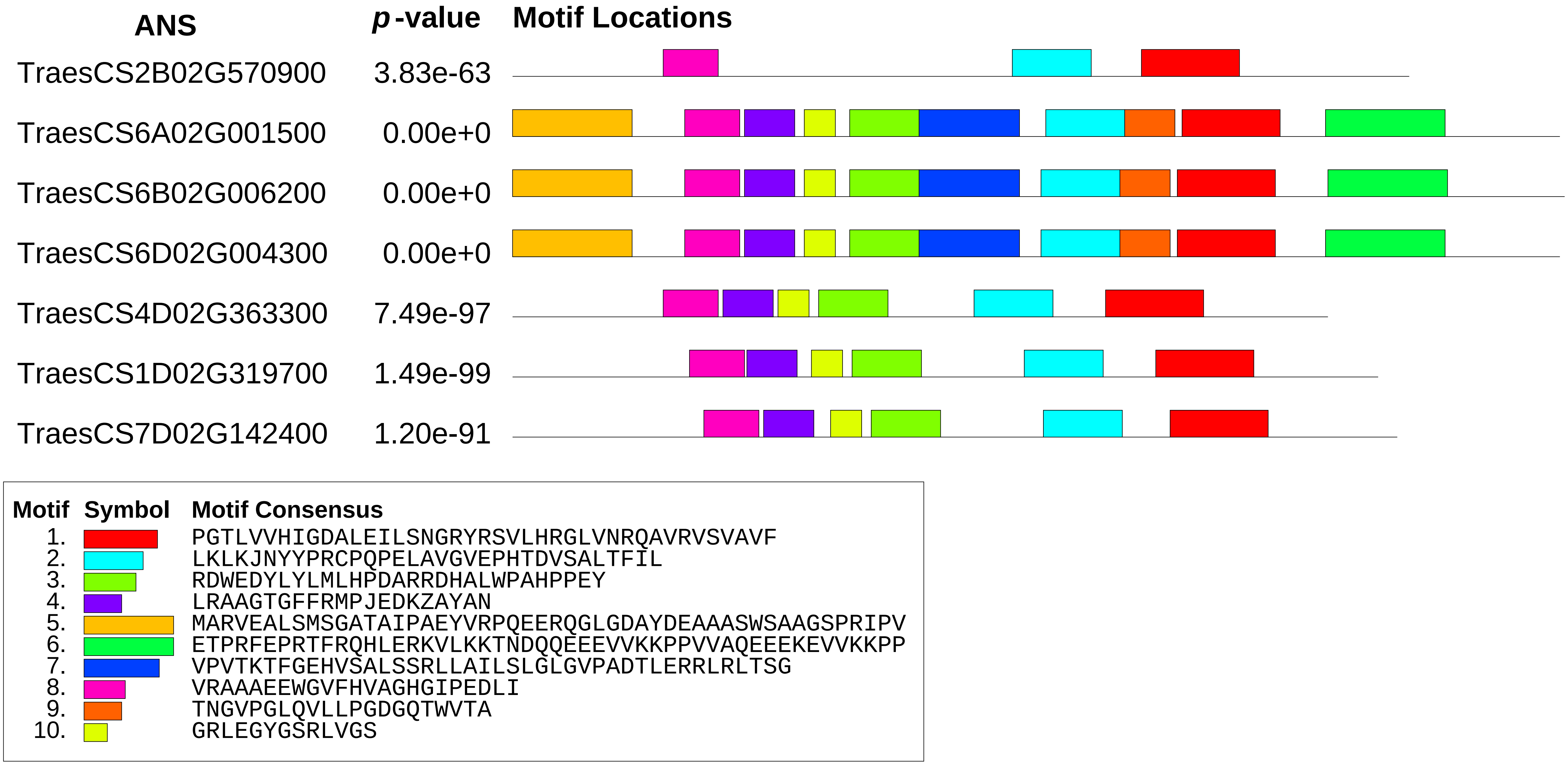

ANS
p-value
Motif Locations
TraesCS2B02G570900 3.83e-63
TraesCS6A02G001500 0.00e+0
TraesCS6B02G006200 0.00e+0
TraesCS6D02G004300 0.00e+0
TraesCS4D02G363300 7.49e-97
TraesCS1D02G319700 1.49e-99
TraesCS7D02G142400 1.20e-91
Motif Symbol Motif Consensus
1. PGTLVVHIGDALEILSNGRYRSVLHRGLVNRQAVRVSVAVF
2. LKLKJNYYPRCPQPELAVGVEPHTDVSALTFIL
3. RDWEDYLYLMLHPDARRDHALWPAHPPEY
4. LRAAGTGFFRMPJEDKZAYAN
5. MARVEALSMSGATAIPAEYVRPQEERQGLGDAYDEAAASWSAAGSPRIPV
6. ETPRFEPRTFRQHLERKVLKKTNDQQEEEVVKKPPVVAQEEEKEVVKKPP
7. VPVTKTFGEHVSALSSRLLAILSLGLGVPADTLERRLRLTSG
8. VRAAAEEWGVFHVAGHGIPEDLI
9. TNGVPGLQVLLPGDGQTWVTA
10. GRLEGYGSRLVGS

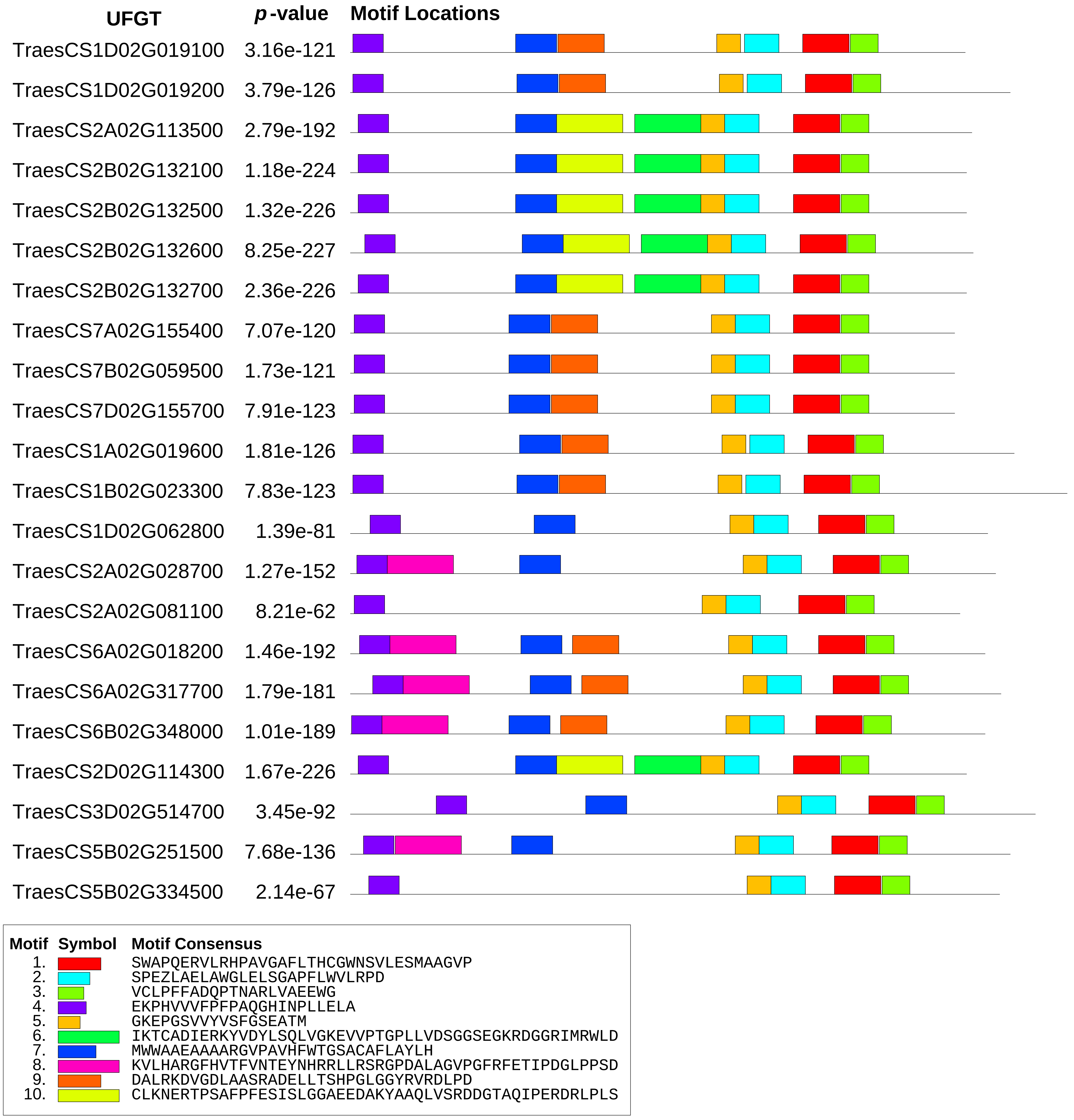
UFGT
p-value
Motif Locations
TraesCS1D02G019100 3.16e-121
TraesCS1D02G019200 3.79e-126
TraesCS2A02G113500 2.79e-192
TraesCS2B02G132100 1.18e-224
TraesCS2B02G132500 1.32e-226
TraesCS2B02G132600 8.25e-227
TraesCS2B02G132700 2.36e-226
TraesCS7A02G155400 7.07e-120
TraesCS7B02G059500 1.73e-121
TraesCS7D02G155700 7.91e-123
TraesCS1A02G019600 1.81e-126
TraesCS1B02G023300 7.83e-123
TraesCS1D02G062800 1.39e-81
TraesCS2A02G028700 1.27e-152
TraesCS2A02G081100 8.21e-62
TraesCS6A02G018200 1.46e-192
TraesCS6A02G317700 1.79e-181
TraesCS6B02G348000 1.01e-189
TraesCS2D02G114300 1.67e-226
TraesCS3D02G514700 3.45e-92
TraesCS5B02G251500 7.68e-136
TraesCS5B02G334500 2.14e-67
Motif Symbol Motif Consensus
1. SWAPQERVLRHPAVGAFLTHCGWNSVLESMAAGVP
2. SPEZLAELAWGLELSGAPFLWVLRPD
3. VCLPFFADQPTNARLVAEEWG
4. EKPHVVVFPFPAQGHINPLLELA
5. GKEPGSVVYVSFGSEATM
6. IKTCADIERKYVDYLSQLVGKEVVPTGPLLVDSGGSEGKRDGGRIMRWLD
7. MWWAAEAAAARGVPAVHFWTGSACAFLAYLH
8. KVLHARGFHVTFVNTEYNHRRLLRSRGPDALAGVPGFRFETIPDGLPPSD
9. DALRKDVGDLAASRADELLTSHPGLGGYRVRDLPD
10. CLKNERTPSAFPFESISLGGAEEDAKYAAQLVSRDDGTAQIPERDRLPLS

Supplemental Table S1: Analysed motifs of anthocyanin biosynthetic genes

	Domain ID	Motif	Function
**CHS**			
1	Chal_sti_synt_C	WNSVFWIAHPGGPAILDMVEAKVNLNKERMRATRHVLSEYGNMSSACVLF	Catalyze the formation of (2S)-5-hydroxyflavanone from 6′-hydroxychalcone (naringenin chalcone)
2	Chal_sti_synt_N	DYQLTKMLGLRPSVKRLMMYQQGCFAGGTVLRLAKDLAENNRGARVLVVC	
3	Chal_sti_synt_N	NCVYQADYPDYYFKITKSDHMADLKEKFKRMCDKSQIRKRYMHLTEEILQ	
4	Chal_sti_synt_C	QLVSASQTILPDSEGAIDGHLREVGLTFHLLKDVPGLISKNIER	
5	Chal_sti_synt_N	PNMCAYMAPSLDARQDIVVVEVPKLGKAAAQKAIKEWGQPRSKITHLVFC	
6	Chal_sti_synt_C	IMDEMRKRSAEDGHATTGEGMDWGVLFGFGPGLTVETVVLH	
7	Chal_sti_synt_N	SEITAVTFRGPHESHLDSLVGQALFGDGAAAVIIGADP	
8	Chal_sti_synt_N	EEVRKAQRAEGPATVLAIGTATPA	
**F3H**			
1	2OG-FeII_Oxy	PPVSGALVVNIGDHLQLMSNGRFKSVEHRAV-VNGEGPRLSVATFF	Role in the 3-hydroxylation of flavanone to form dihydroflavonol
2	2OG-FeII_Oxy	VAVNYYPACPZPDLTLGLKRHTDPGFJTLLLQDA	
3	DIOX_N	CETWGFFQVVNHGVPEALIADMLRAVREF	
6	MbtH	WPEK---PAGWRAVVER	
9	BNR	ATRDGGKTW	
**F3’H**			
1	p450	PRESQEDCQVQGYDVPAGTRVLVNAWAIGRDPAYWEDAEEF	Catalyze the hydroxylation step to impart purple-blue pigments
2	p450	FKGNDFZFLPFGAGRRMCPGMAFG	
3	p450	IKAVJLDVFAAGTDTSATTJEWAMAELMRNPRVMQKAQAEV	
5	p450	EDDLAKLPYLKAVIKETLRLHPPAPL	
6	p450	VPTVVVSSAEAAREVLKTHDHVFASRP	
**F3’5’H**			
1	p450	YLQAVIRETLRLHPYGPLJPPREAMEDCTVAGYDVPRGTQVIVNVWALGR	Catalyze the hydroxylation step to impart purple-blue pigments
2	p450	IKAIVLDILIAGTDTSATTVEWAMAELMNNPEVMRKATAELDAVVG	
4	p450	FLPFGAGRRGCPGIALGLRFVGLALASLLQGFDW	
**DFR**			
1	GDP_Man_Dehyd	VTGGGGFIGSWJVKLLLDRGYTVHGTVRNPQDEK	

**1**	Epimerase	VCVTGGGGFIGSWJVKLLLDRGYTVHGTVRNPQDEKNA	NAD dependent epimerase/dehydratase
**2**	NAD_binding_4	KTELWYCVSKTLAEKAAWEFAGEEGLELVVVNPGLVLG	
**3**	NAD_binding_10	DERLRLFQADLLDPASLRAAIEGCHGVFHLA	
**3**	Epimerase	DERLRLFQADLLDPASLRAAIE--GCHGVFHLAS	
**6**	3Beta_HSD	GTLNVLRAAKDAGVKRVVMTSSQTAVCPN	
**ASN**			
**1**	2OG-FeII_Oxy	GTLVVHIGDALEILSNGRYRSVLHRGL-VNRQAVRVSVAVF	Catalyze a remarkably diverse range of oxidative reactions
**2**	2OG-FeII_Oxy	KJNYYPRCPQPELAVGVEPHTDVSALTFIL	
**8**	DIOX_N	VRAAAEEWGVFHVAGHGIPEDLI	
**UFGT**			
**1**	UDPGT	WAPQERVLRHPAVGAFLTHCGWNSVLESMAAGVP	UDP-glycosyltransferase
**2**	UDPGT	VCLPFFADQPTNARLVAEEWG	
